# A potential therapeutic strategy for chronic lymphocytic leukemia by combining Idelalisib and GS-9973, a novel spleen tyrosine kinase (Syk) inhibitor

**DOI:** 10.18632/oncotarget.1484

**Published:** 2014-10-30

**Authors:** Russell T Burke, Sarah Meadows, Marc M Loriaux, Kevin S. Currie, Scott A. Mitchell, Patricia Maciejewski, Astrid S. Clarke, Julie A. Dipaolo, Brian J. Druker, Brian J. Lannutti, Stephen E. Spurgeon

**Affiliations:** ^1^ Knight Cancer Institute, Oregon Health & Science University, Portland, OR; ^2^ Gilead Sciences Inc, Seattle, WA; ^3^ Gilead Sciences Inc, Branford, CT; ^4^ Division of Pathology, Oregon Health & Science University, Portland, OR; ^5^ Howard Hughes Medical Institute, Bethesda, MD; ^6^ Division of Hematology and Medical Oncology, Oregon Health & Science University

**Keywords:** Chronic Lymphocytic Leukemia, B-cell receptor, signaling pathways, spleen tyrosine kinase, PI3 kinase

## Abstract

Agents that target B-cell receptor (BCR) signaling in lymphoid malignancies including idelalisib (GS-1101) and fostamatinib which inhibit the delta isoform of PI3 kinase (PI3Kd) and spleen tyrosine kinase (Syk) respectively have shown significant clinical activity. By disrupting B-cell signaling pathways, idelalisib treatment has been associated with a dramatic lymph node response, but eradication of disease and relapse in high risk disease remain challenges. Targeting the BCR signaling pathway with simultaneous inhibition of PI3Kd and Syk has not yet been reported. We evaluated the pre-clinical activity of idelalisib combined with the novel and selective Syk inhibitor GS-9973 in primary peripheral blood and bone marrow Chronic Lymphocytic Leukemia (CLL) samples. Both PI3Kd and Syk inhibition reduced CLL survival and in combination induced synergistic growth inhibition and further disrupted chemokine signaling at nanomolar concentrations including in bone marrow derived and poor risk samples. Simultaneous targeting of these kinases may significantly increase clinical activity.

## INTRODUCTION

Chronic lymphocytic leukemia (CLL) remains incurable with standard therapies [1]. Increasingly it has been recognized that CLL cell survival is dependent on the complex interactions of neoplastic cells with the microenvironment. Specifically, cell proliferation is mediated by multiple inputs such as B-cell receptor (BCR) signaling pathways, chemokines, cytokines, integrins, nurse-like cells, BAFF, and CD40 [2]. A number of therapeutic agents have been developed including small molecules that inhibit these key signaling pathways including agents that target BCR-mediated kinase signaling pathways and disrupt CLL cell-microenvironment interactions [3,4]. Treatment of primary CLL cells with idelalisib (GS-1101), ibrutinib, and fostamatinib (R406) which inhibit the PI3 kinase delta-specific isoform (PI3Kd), Bruton's tyrosine kinase (Btk) and spleen tyrosine kinase (Syk) respectively, results in inhibition of BCR signaling pathways, decreased cell proliferation, and disruption of chemokine mediated CLL cell migration [5],[6],[7],[8]. These agents are orally bioavailable and have been evaluated in early phase trials in relapsed and refractory CLL patients. Although significant clinical activity has been observed in patients treated with these drugs as single agents, complete remission rates are low and marrow disease may be difficult to eradicate. Furthermore, although response rates with monotherapy do not appear to be adversely affected by the presence of poor risk disease, responses are significantly less durable in this population [9, Brown J.R. et al. J Clin Oncolo 31, 2013 (supple;abstr 7003)]. Thus new therapeutic approaches that evaluate these agents in combination are warranted.

Currently, little is known about the effects of inhibiting multiple nodes in the BCR pathway. Feedback loops and cross talk between signaling pathways may significantly impact the efficacy of cancer therapeutics and drive resistance to single agent therapy. Combination therapy to address the molecular complexity associated with the convergence of B-Cell signaling pathways could provide a novel treatment approach. Inhibition of multiple B-cell signaling pathways and simultaneous inhibition of the BCR signaling pathway may have the potential for synergy and implications for overcoming resistance to single agents or eradicating minimal residual disease (MRD) the latter of which has been shown to correlate with survival after chemo-immunotherapy [10]. These considerations prompted us to assess the effects of dual PI3Kδ and Syk inhibition in CLL using idelalisib and the novel Syk inhibitor GS-9973.

## RESULTS

### The Combination of Idelalisib and GS-9973 Synergistically Inhibits Cell Viability at Nanomolar Concentrations in vitro

**Figure 1:**
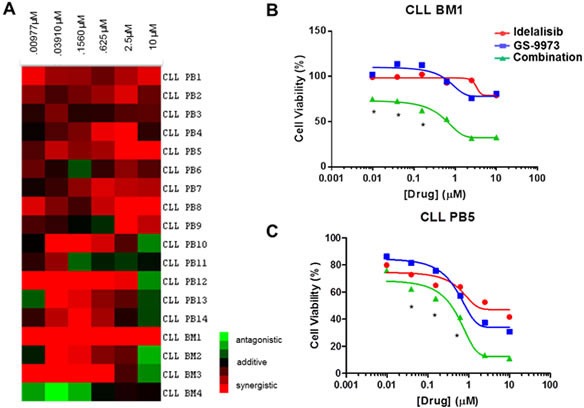
(A) A heat map displaying interaction indices at individual concentration points for each CLL sample. R values >1 (antagonistic), 1 (additive) and <1 (synergistic) are color coded as green, black and red, respectively. Specifically, the heat map was created using TreeView by transforming the interaction index for each concentration point of the primary samples by log_2_. Each row represents an individual sample. Peripheral blood samples are labeled “PB” and bone marrow samples are labeled “BM.” The columns represent (from left to right) increasing equimolar concentrations of each drug drug ranging from .977 nM to 10 μM. (B) A representative viability curve for CLL BM1 is shown with viability on the y axis and increasing equimolar concentrations of drug (μM) on the x axis. Resistance to each agent as monotherapy is seen (top 2 curves), an effect that is overcome by the drug combination (bottom curve)when the combination is used. (C)) Viability curve for a sample representative of having synergy. Although all concentrations of the drug combination were synergistic, (CI < 1), some concentrations had values that lost significance when accounting for the 95% confidence interval. Therefore, asterisks are provided which denote points that are significantly synergistic within the 95% confidence interval as defined by R. Idelalisib (GS-1101) treated cells are shown in red, GS-9973 in blue, and the combination in green.

Significant synergy was seen in the majority of samples treated with idelalisib and GS-9973. A heat map (Figure 1A) of plotted interaction indices depicting the sensitivity to the combination is shown. For the majority of samples without synergistic responses, additive interactions were observed. Specific disease and/or biologic characteristics are shown in Table 1. Half of all samples were obtained from patients with relapsed disease and two of four bone marrow derived samples were from patients with refractory disease. Three samples harbored a 17p deletion and seven samples had an unmutated variable region of the immunoglobulin heavy chain (IgVh). Disease factors, such as relapsed/refractory disease, IgVh mutational status, and fluourescent in situ hybridization results (FISH) did not correlate with achieving a synergistic response. However, notably, of the 3 samples (PB4, PB14, and BM3) harboring a17p deletion synergy was seen. Representative cell viability curves for individual samples are shown (Figure 1B and 1C). Data for all single agent and combination viability curves are also included (Supplemental Figure 1). Three of four bone marrow derived samples showed synergy. Interestingly, CLL BM1 was resistant (i.e. no significant decrease in cell viability) to each drug alone but synergistically sensitive to the combination.

**Table 1:** Disease status and biologic CLL disease characteristics

Sample ID	Disease Status	Cytogenetics/FISH	IgVh mutational status	ZAP 70	CD38
PB 1	Relapsed	Normal	Mutated	Negative	Negative
PB 2	Untreated	Normal	Mutated	Negative	Negative
PB 3	Relapsed	Unknown	Unknown	Unknown	Unknown
PB 4	Relapsed	17p deletion 13q deletion	Unmutated	Positive	Positive
PB 5	Untreated	13q deletion	Mutated	Negative	Negative
PB 6	Untreated	13q deletion	Mutated	Unknown	Unknown
PB 7	Untreated	13q deletion	Mutated	Negative	Negative
PB 8	Untreated	13q deletion	Mutated	Negative	Negative
PB 9	Relapsed	13q deletion	Unknown	Unknown	Unknown
PB 10	Relapsed	11q deletion 13q deletion	Unmutated	Negative	Positive
PB 11	Untreated	13q deletion	Mutated	Unknown	Unknown
PB 12	Relapsed	Trisomy 12	Unmutated	Positive	Negative
PB 13	Unknown	Unknown	Unknown	Unknown	Unknown
PB 14	Refractory	Complex 17p deletion Trisomy 12	Unmutated	Positive	Negative
BM 1	Refractory	13q deletion	Unmutated	Positive	Positive
BM 2	Relapsed	13q deletion Trisomy 12	Umutated	Positive	Negative
BM 3	Refractory	17p deletion 13q deletion	Unknown	Negative	Negative
BM 4	Untreated	13q deletion	Unmutated	Negative	Negative

### Idelalisib and GS-9973 Inhibit BCR mediated signaling pathways

**Figure 2:**
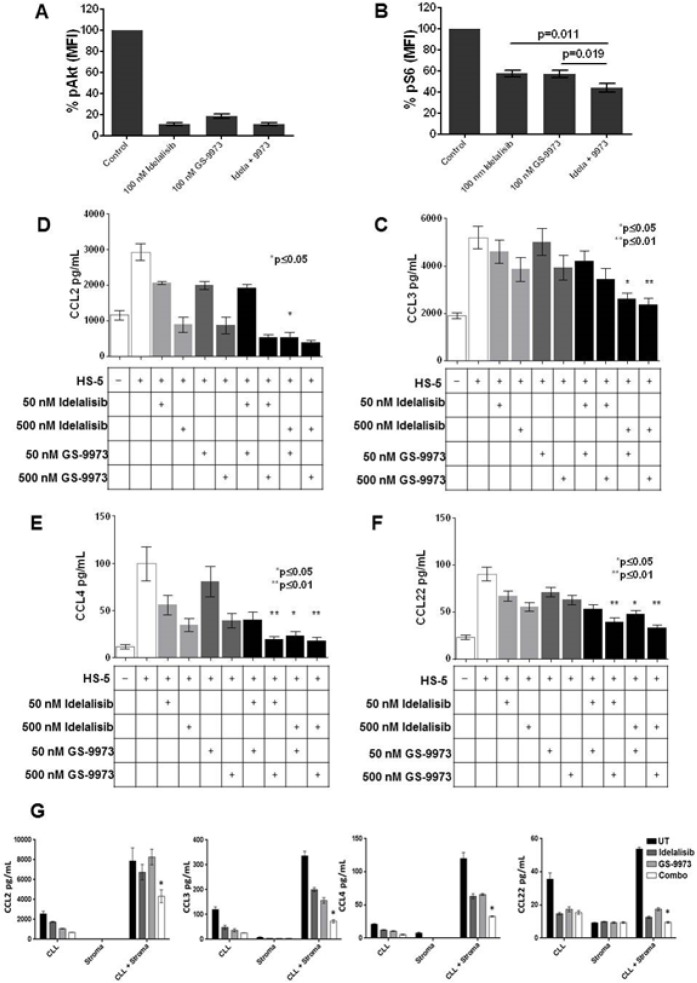
Treatment of primary CLL cells (n =14) co-cultured with HS5 stromal cells with idelalisib (100 nM) or GS-9973 (100 nM), alone or in combination (100 nM each), results in decreased AKT phosphorylation (A) while treatment with the combination significantly decreases S6 phosphorylation as compared to treatment with each drug alone (p = 0.011 for the combination compared to GS-1101 treatment alone and p= 0.019 compared to GS-9973 alone). Each condition was compared using a two tailed t-test. (B)The effect of idelalisib (GS-1101) and/or GS-9973 on CCL2, CCL3, CCL4, and CCL22 expression after CLL-HS-5 co-culture. The bar diagrams represent the mean supernatant concentrations (+/− SEM) of CCL2 (C), CCL3 (D), CCL4 (E), CCL22 (F) from CLL cells co-cultured with or without (controls) HS-5 cells and various concentrations of idelalisib, GS-9973, or a combination of idelalisib and/GS-9973 (50 nM plus 50 nM, 50 nM plus 500 nM for each drug, and 500 nM plus 500 nM for each drug) in 6 different patient samples assessed after 24 hours. Chemokine expression after treatment with individual drug or the combination was compared using a two tailed t-test. The combination of idelalisib and GS-9973 showed significant effect on decreasing CCL2, CCL3, CCL4, CCL22. A single asterisk denotes significant (p < .05) and a double asterisk (**) represents a significance of p < .01. Differences between single agent treatment and the combinations are shown. Panels G-J show increased chemokine expression when CLL cells are cultured in the presence of primary bone marrow derived stromal cells (n=3). Similar to the HS5 co-culture system, co-culture with primary bone marrow leads to increased chemokine expression (mean +/− SEM supernatant concentrations) which is abrogated by treatment with idelalisib (100 nM) and GS-9973( 100 nM) for 24 hours. As shown in the last column in each figure, this effect that is significantly enhanced by combining these two agents (p < .05, two tailed t-test).

To confirm that idelalisib and GS-9973 effectively inhibit BCR mediated signaling, we evaluated phosphorylation of Akt and ribosomal S6 in CLL cells after treatment with idelalisib and GS-9973. Treatment with each drug alone significantly decreased pAkt at nanomolar concentrations while combination treatment did not result in a significant further decline in pAkt when compared to each inhibitor alone. Conversely, although single agent treatment with idelalisib and GS-9973 decreased S6 phosphorylation, the combination was significantly more potent (Figure 2B). To further evaluate these agents ability to inhibit BCR mediated signaling after IgM stiumulation, using immunoblotting, we also evaluated the inhibitory effects on Ramos cells, a Burkitt's cell line with an intact BCR. A significant decrease in AKT phosphorylation was seen with each drug alone while ERK phosphorylation was inhibited by GS-9973 +/− GS-1101. S-6 phosphorylation was also inhibited by the combination (data not shown).

### Effects of Combination of Chemokine/Cytokine Networks

When CLL cells were co-cultured with HS5 stromal cells (n=6), significantly higher levels of CCL2, CCL3, CCL4 and CCL22 were seen (Figure 2). Combination treatment significantly decreased CCL2 (2C), CCL3 (2D), CCL4 (2E), CCL22 (2F) compared to each individual drug. To validate the results seen using the HS-5 co-culture system, we also tested the effects of idelalisib or GS-9973 on CLL cells cultured in primary BM stromal cells (n=3) and saw significant decreases in chemokine levels (Figure 2G) after idelalisib plus GS-9973 treatment.

## DISCUSSION

Here we show that the combination of idelalisib and GS-9973 synergistically decreases CLL cell viability in the majority of CLL samples tested. Significant growth inhibition was seen in the presence of HS-5 conditioned media and in patient derived primary bone marrow CLL cells, which are known to have an activated phenotype [13] and be more resistant to cytotoxic therapy [14], [15]. Interestingly one bone marrow derived sample that was resistant to each drug as a single agent demonstrated significant growth inhibition when treated with idelalisib and GS-9973 in combination. This suggests that this difficult to eradicate disease compartment may potentially be successfully targeted with simultaneous PI3Kd and Syk inhibition. This may not only enhance treatment efficacy but also suggests a role for treating minimal residual disease, the presence of which is associated with treatment failure after initial therapy [16].

As drugs that target the BCR are not classic cytotoxic agents, their ability to inhibit their downstream targets and disrupt chemokine/cytokine interactions, which are essential for CLL cell homing and survival, are critical to their mechanism of action. Akt and S6 are known to be regulated by PI3K and Syk which are effected by BCR activation [17],[18]. PI3K also receives co-stimulatory signals from BAFF, CD40, and toll-like receptor pathways while Fc receptors, chemokines, and integrins can co-stimulate Syk activation [2]. Phospho-Akt (pAkt) has previously been shown to decrease after idelalisib treatment [19] and with Syk inhibition [20]. Here we confirm that treatment of primary CLL cells with idelalisib decreases pAkt. GS-9973 also inhibits pAkt at nanomolar concentrations. Combination treatment did not result in a significant further decline in pAkt when compared to each inhibitor alone. The finding that the combination's ability to further inhibit S6 phosphorylation is not surprising as S6 lies downstream of multiple kinase pathways and again highlights the potential efficacy of inhibiting two tightly integrated yet distinct signaling pathways.

Chemokine and cytokine levels have been shown to be elevated in CLL and are known to be an integral part in maintenance of the CLL cell microenvironment interaction [21]. Increased expression of CCL3 and CCL4 has been seen in CLL after BCR activation or co-culture with nurse like cells (NLCs) and may contribute to recruitment of T-cells [22]. Recruitment of T-cells is further augmented by CCL22 production while CCL2 has been shown to augment CLL cell survival in vitro [23]. In addition, stromal cell chemokines, such as CXCL12, aid in CLL chemotaxis into the stromal compartment and activation of pro-survival signaling. This effect has been shown to be abrogated by treatment with a number of agents that target BCR mediated pathways including Syk [25]. In addition, it is well established that patients treated with idelalisib have significant reductions in CCL3 and CCL4 and disruption of these networks results in significant migration of CLL cells out of lymph nodes. Notably, we found that when CLL cells were cultured with HS-5 stromal cells or primary bone marrow cells, increases in CCL2, CCL3, CCL4, and CCL22 were seen. Interestingly, after treatment with each drug alone, inhibition of chemokine expression was observed an effect that was significantly enhanced when using idelalisib and GS-9973 in combination at nM concentrations. These data suggest that using this combination can further disrupt chemokine networks known to be essential to CLL homing and survival. These findings may have implications for treating resistance to chemo-immunotherapy, single agent kinase inhibitors, or for targeting minimal residual disease especially in poor risk patients who have significantly shorter remissions with monotherapy.

We have shown that the combination of idelalisib and the novel Syk inhibitor, GS-9973, is effective at synergistically decreasing CLL cell viability, inhibiting BCR mediated signaling, and disrupting chemokine expression in CLL. These data provide rationale for the clinical combination of agents including PI3Kd and Syk inhibitors in CLL that target multiple B-cell signaling pathways and simultaneously target BCR mediated pathways. Such Informed combinations, directed at bypassing feedback loops and interrupting cross talk between signaling pathways may improve therapeutic outcomes and continue to significantly inform our approach to CLL treatment.

## MATERIALS AND METHODS

### Cell isolation and culture

After informed consent, in accordance with the Declaration of Helsinki and approved by the institutional review board, lymphocytes were isolated from the peripheral blood (n=14) or bone marrow (n=4) of CLL patients and purified using a Ficoll-Paque PLUS (GE Healthcare) gradient. Disease status and biologic characteristics for each sample was recorded. Cells were used fresh or collected for viable frozen samples in fetal bovine serum with 10% DMSO and stored in liquid nitrogen. Culture media was made with RPMI-1640 (Gibco) supplemented with 10% fetal bovine serum, 4mM L-Glutamine, penicillin and streptomycin. Conditioned media was prepared by culturing the RPMI over confluent HS-5 human stromal cell line for 3 hours.

### Inhibitor combinations and synergy

Ficoll purified CLL cells (2x10^5^) isolated from bone marrow or peripheral blood were treated with each drug alone and with six equimolar concentrations of idelalisib and GS-9973-obtained from Gilead Sciences, Inc. ranging from .977 nM to 10μM on 96-well plates in triplicate. GS-9973 is highly selective for Syk. Its chemical structure and kinase selectivity using the DiscoveRx kinome panel (San Diego, CA) for Kd determinations as compared to R406 are shown in Table 2. Plated cells were then cultured in HS-5 conditioned media at 37°C with 5% CO_2_. After 72 hours of culture, viability was determined using an MTS assay (Cell Titer 96, Promega). Viability data were used to generate cell viability curves for each drug alone and in combination for each sample. The potential synergy of the combination of idelalisib and GS-9973 at a given equimolar concentration was determined using the median effect model [11]. The statistical modeling was run in R using a script that utilizes the median effect model [12]. A value of 1, less than 1, and greater than 1 using R defines an additive interaction, synergistic and antagonistic, respectively. The Lee et al. method calculates a 95% confidence interval for each data point. For each viability curve, to be considered synergistic, a data point must have an interaction index below 1 and the upper confidence interval must also be below 1. In order to summarize and demonstrate collective synergy results, a heat map was created using TreeView by transforming the interaction index for each concentration point of the primary samples by log_2_.

**Table 2:** Chemical characterization of GS-9973 Panel A shows the chemical structure for GS-9973. Panel B shows the relative selectivity of GS-9973 compared to the Syk inhitor R406 using the DiscoveRx panel for Kd determinations

**Figure:** 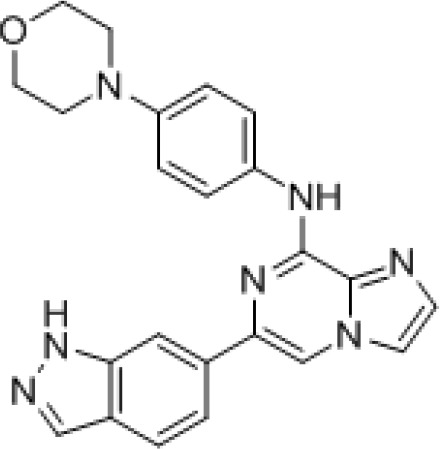 **Table:** Kinome Scan Symbol GS-9973 R406 Kd (nM) Selectivity (Fold) Kd (nM) Selectivity (Fold) ABL1-nonphos. 8400 800 8400 560 ABL1-phos. 560 53 560 37 ALK 2900 276 240 16 AXL 1900 181 82 5 BIKE 1100 105 1900 127 BLK 420 40 25 1.7 BRK 330 31 360 24 CHEK2 40000 3810 1000 67 CSF1R 180 17 31 2.1 CSNK2A1 540 51 1400 93 CSNK2A2 110 10 1200 80 DAPK1 40000 3810 55 4 DAPK3 40000 3810 13 0.9 EGFR 1400 133 300 20 EPHA1 470 45 67 4 EPHB6 550 52 310 21 FGR 230 22 33 2.2 FLT3 320 30 0.71 0.05 FLT3-autoinhib. 40000 3810 13 0.9 GAK 1300 124 250 17 GRK4 40000 3810 450 30 HCK 410 39 150 10 HIPK1 40000 3810 820 55 HIPK3 40000 3810 3200 213 INSR 1800 171 1100 73 IRAK1 140 13 9.7 0.6 IRAK3 120 11 11 0.7 JAK1(JH1-cat.) 730 70 21 1.4 JAK2(JH1-cat.) 110 10 3.5 0.2 JAK3(JH1-cat.) 3200 305 36 2.4 JNK1 2000 190 38 3 KIT 210 20 6.8 0.5 LCK 820 78 30 2.0 LOK 40000 3810 70 5 LTK 1200 114 800 53 MA P4K2 40000 3810 670 45 MEK5 200 19 27 1.8 MERTK 3300 314 170 11 MLK1 570 54 4.3 0.3 PDGFRB 670 64 3.3 0.2 PIP5K1A 40000 3810 1900 127 PIP5K1C 40000 3810 PIP5K2B 40000 3810 1200 80 PRKR 3000 286 180 12 RET 1000 95 4.1 0.3 RIOK1 40000 3810 340 23 RIOK2 160 15 3400 227 RIOK3 40000 3810 210 14 SBK1 40000 3810 93 6 SLK 820 78 33 2.2 SRC 110 10 8.4 0.6 SRMS 910 87 610 41 SRPK1 40000 3810 590 39 SRPK3 3200 305 400 27 STK16 330 31 1.7 0.1 STK33 40000 3810 970 65 **SYK** **10.5** **1** **15** **1** TAK1 490 47 49 3 TGFBR2 390 37 330 22 TNK1 86 8 25 1.7 TNK2 670 64 70 5 TYK2(JH1-cat.) 180 17 190 13 ULK3 510 49 7.1 0.5 VEGF R2 40000 3810 40 3 YES 450 43 35 2.3 YSK4 440 42 67 4 ZAP70 410 39 21 1.4

### Inhibition of BCR mediated signaling pathways

For flow cytometry experiments, ficoll purified CLL cells obtained from 14 patients were co-cultured with HS5 stromal cells and then treated with each drug alone (100 nM) or in combination (100 nM each) for 24 hours. CLL cells were stained with anti-CD5-fluorescein isothiocyanate (FITC) (Clone BL1a, Beckman Coulter, Indianapolis IN) and anti-CD19- Phycoerythrin (PE) (Clone 1D3, BD Biosciences, San Jose CA) to identify CLL cells, and either anti- phopho-Akt^S473^, anti-phospho-S6 (Alexa Fluor 488, Life Technologies, Carlsbad, CA) or an isotype-matched control antibody (mouse IgG1-Alexa Fluor 488 conjugate, Cell Signaling, Danvers, MA). FITC-CD5/CD19^+^ cells were gated (using anti-CD5-FITC and Anti-CD19-PE) and analyzed by 3-color flow cytometry to quantify intracellular phospho-Akt^S473^ levels (Cytomics FC 500MPL cell sorter and MXP Version 2.2 software, Beckman Coulter, Brea, CA).

To further validate these agents' ability to inhibit BCR mediated signaling we evaluated Ramos cells, a Burkitt's cell line with intact BCR. 5x10^6^ cells were plated and treated with 1 μM of idelalisib, GS-9973 alone or in combination and were cultured for 24 hours in standard culture media. 20μg Goat F(ab')_2_ anti-human IgM (Invitrogen) was added to stimulate the BCR for 30min. Protein isolates were subjected to immune-blotting with Phospho-Akt (S473), phospho-Erk (T202/Y204) (Cell Signaling), and phospho-S6 (Cell signaling).

### Chemokine expression in HS-5 co-culture or primary bone marrow

The effect of idelalisib and/ or GS-9973 treatment on chemokine expression after CLL- HS-5 co-culture (n=6) or CLL co-culture with primary bone marrow derived CLL cells (n=3) was measured after 24 hours of incubation with individual drug (50 nM or 500 nM) or in combination using four conditions (50 nM plus 50 nM, 50 nM plus 500 nM for each drug, and 500 nM plus 500 nM for each drug). For the marrow samples, ficoll purified frozen CLL samples from the same patient were thawed and 5x10^6^ cells added to marrow stromal cells and incubated with each drug alone or in combination in triplicate. Supernatants were harvested and assayed for CCL2, CLL3, CCL4, and CCL22 by quantitative ELISA (Quantikine; R&D Systems). A two tailed t-test was used to compare the effect of drug combinations compared to treatment with each individual drug.

## SUPPLEMENTARY FIGURE


