# Supplementary material for: A potential therapeutic strategy for chronic lymphocytic leukemia by combining Idelalisib and GS-9973, a novel spleen tyrosine kinase (Syk) inhibitor
Source: Oncotarget. 2014 Oct 30;5(4):908–15. doi: 10.18632/oncotarget.1484 (PMC4011593; doi:10.18632/oncotarget.1484)
Supplement: Supplementary file 1 [file oncotarget-05-908-s001.pdf]

A potential therapeutic strategy for chronic lymphocytic leukemia by combining Idelalisib and GS-9973, a novel spleen tyrosine kinase (Syk) inhibitor - Burke et al

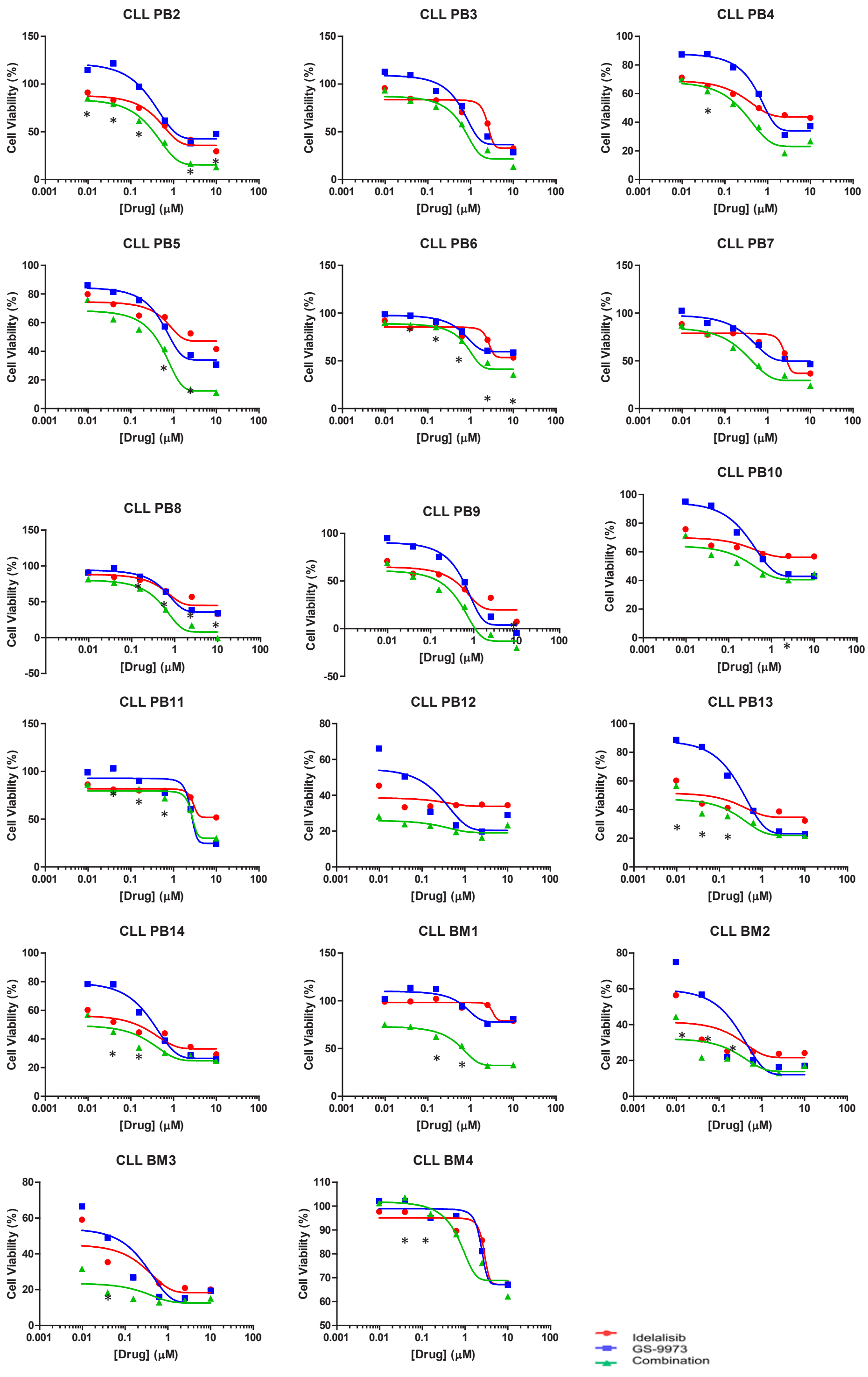

Supplemental Figure 1: Ficoll purified CLL cells ($2x10^5$) obtained from the peripheral blood of 14 patients and the bone marrow of 4 patients were treated in triplicate with 6 concentrations (.977nM to 10µM) of idelalisib, GS-9973, or equimolar concentrations of each drug in combination. Viability (y axis), determined after 72 hours of culture with drug in HS-5 conditioned media, is plotted against increasing drug concentrations (x axis). Each graph represents an individual sample. Asterisks denote synergistic concentrations as determined using the 95% confidence interval as defined by R.
